# Monoclonal antibody humanness score and its applications

**DOI:** 10.1186/1472-6750-13-55

**Published:** 2013-07-05

**Authors:** Sean H Gao, Kexin Huang, Hua Tu, Adam S Adler

**Affiliations:** 1 LakePharma, Inc., 530 Harbor Boulevard, Belmont, CA 94002, USA

**Keywords:** Therapeutic antibody, Humanization, Immunogenicity

## Abstract

**Background:**

Monoclonal antibody therapeutics are rapidly gaining in popularity for the treatment of a myriad of diseases, ranging from cancer to autoimmune diseases and neurological diseases. Multiple forms of antibody therapeutics are in use today that differ in the amount of human sequence present in both the constant and variable regions, where antibodies that are more human-like usually have reduced immunogenicity in clinical trials.

**Results:**

Here we present a method to quantify the humanness of the variable region of monoclonal antibodies and show that this method is able to clearly distinguish human and non-human antibodies with excellent specificity. After creating and analyzing a database of human antibody sequences, we conducted an in-depth analysis of the humanness of therapeutic antibodies, and found that increased humanness score is correlated with decreased immunogenicity of antibodies. We further discovered a surprisingly similarity in the immunogenicity of fully human antibodies and humanized antibodies that are more human-like based on their humanness score.

**Conclusions:**

Our results reveal that in most cases humanizing an antibody and confirming the humanness of the final form may be sufficient to eliminate immunogenicity issues to the same extent as using fully human antibodies. We created a public website to calculate the humanness score of any input antibody sequence based on our human antibody database. This tool will be of great value during the preclinical drug development process for new monoclonal antibody therapeutics.

## Background

The first monoclonal antibody therapeutic was approved for use in the United States in 1986, the mouse anti-CD3 molecule muromonab that reduces organ transplant rejection [1]. Since then, nearly 30 full-length therapeutic antibodies and 10 fragment antigen-binding (Fab) antibodies have been approved in the United States or Europe, with hundreds more in preclinical and clinical development [2-7]. Therapeutic antibodies are used in a myriad of disease settings, including oncology, autoimmune, and neurological disorders. There are four major antibody types used as therapeutics: fully non-human (most commonly mouse), chimeric (non-human variable region, human constant) [8], humanized (human and non-human variable region sequence, human constant) [9,10], and fully human [4]. Therapeutics have evolved to include more human sequences, since in general the more human sequence that is present in the antibody, the less immunogenic the antibody will be once introduced to humans [11]. Immunogenicity refers to the ability of a therapeutic antibody to induce the formation of anti-drug antibodies (ADAs) when administrated into a human. ADAs are immune system generated antibodies against the therapeutic that can reduce the efficacy of the drug, and more importantly they can also cause adverse effects ranging from a rash at the site of injection to a systemic inflammatory reaction that can be fatal [12,13]. Therefore it would be extremely valuable to know whether an antibody may be immunogenic prior to clinical trials are initiated.

The complementarity determining regions (CDRs) of an antibody are the most variable segments of the variable domain that are essential for the antibody-antigen binding specificity and affinity [14]. The CDRs and the surrounding framework segments comprise the full variable sequence of an antibody. Several attempts have previously been made to calculate a humanness score of the variable region sequences of antibodies, since a vital use for such a tool is to aid in identifying antibody sequences that are likely to have reduced immunogenicity when introduced to humans. First, Abhinandan and Martin [15] developed the H-score, which calculates the average sequence identity of a given antibody sequence as compared to a small database of human variable region sequences. Using these data, however, the authors were unable to find a clear correlation between H-score and immunogenicity of a small set of therapeutic antibodies. Second, Pelat et al. [16] defined a germinality index that is the proportion of framework residues that are identical between a given antibody variable region sequence and the closest related human germline sequence. The score was used to analyze humanized forms of a macaque anthrax toxin antibody. Third, Thullier et al. [17] developed the G-score, which is similar to the H-score expect that the score attempts to classify which germline framework sequence the input antibody was originally derived from. The authors used this tool to study the humanness of multiple macaque antibody sequences.

The germinality index only compares sequences to a single germline sequence, and the databases for the H-score and G-score were composed of a small set of ~3,500 antibody sequences. Further, after assigning H-scores to a set of mouse antibody sequences, nearly all mouse heavy chain sequences scored in the same range as human heavy chain sequences, and more than half of the mouse light chains scored in the same range as human light chain sequences [15]. If an antibody humanness scoring method is unable to clearly distinguish a human and mouse sequence, it is not possible to definitely state how human a given sequence really is by these scoring methods. Here we have developed a new tool to determine the humanness of antibody variable region sequences, termed the T20 score analyzer. After refining the input antibody databases, we found that the T20 score can clearly separate human sequences from mouse sequences and many other species as well. The T20 score was used to conduct a thorough analysis of a large set of therapeutic antibodies, revealing surprising similarities between human-like humanized antibodies and fully human antibodies. The T20 score analyzer is available for free use online: http://abAnalyzer.lakepharma.com.

## Results

### T20 score analyzer development

**Figure 1:**
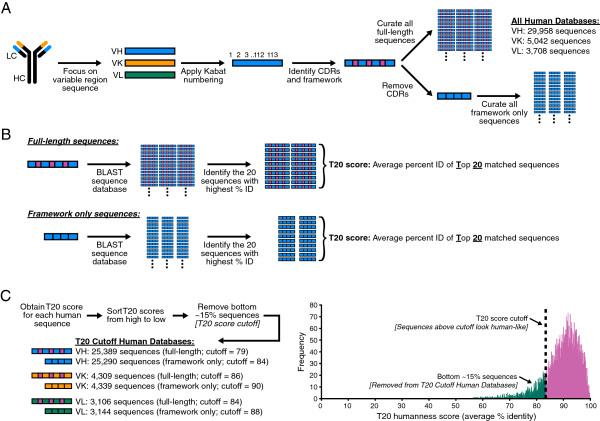
**T20 analyzer development. (A)** Antibody sequence curation. Variable region protein sequences had Kabat numbering applied and the CDR regions identified. Variable heavy chain (VH), variable light kappa chain (VK), and variable light lambda chain (VL) sequences were curated into databases as full-length sequences or framework-only sequences (where the CDR regions were removed). The number of unique antibody sequences in each database is shown. **(B)** Defining the T20 score. Protein BLAST is used to determine the sequence identity of an input sequence with each database sequence. The percent identities of the Top 20 matched sequences are averaged to obtain the T20 score for a given input sequence. **(C)** Left: Details of obtaining the T20 Cutoff Human Databases. Right: Histogram of the T20 scores of a sample set of antibody sequences, indicating the T20 score cutoff and the 15% of sequences below this cutoff that were removed to form the T20 Cutoff Human Database for each chain type. The number of unique antibody sequences in each database is shown.

**Figure 2:**
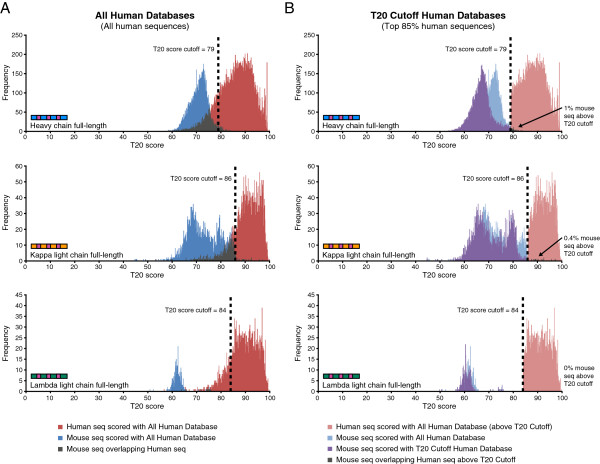
**T20 scores distinguish human and mouse antibody variable region sequences. (A)** T20 scores using All Human Databases. Shown are histograms of the T20 scores for large sets of human or mouse antibody sequences of the indicated chain type. The T20 score cutoff for each antibody chain is indicated by the dashed line. **(B)** Comparing scores using T20 All Human and Cutoff Human Databases. Shown are histograms of the T20 scores for the same set of human or mouse antibody sequences scored with the indicated database. Note that the human sequences with scores below the T20 score were removed from these graphs. The percent of mouse antibodies sequences scored with the T20 Cutoff Human Database that are above the T20 cutoff is provided on the right.

First, we curated a large database of ~38,700 human antibody variable region sequences in order to develop a new tool to analyze human antibody variable region sequences. The variable region sequence of antibody sequences were numbered using the Kabat numbering system [18] and the CDR and framework regions were annotated. Sequences for variable heavy chain (VH), variable kappa light chain (VK), and variable lambda light chain (VL) were stored as full-length sequences or as framework only sequences (where the CDR regions were removed from the sequences) to obtain the All Human Databases (Figure 1A). Starting with a single input variable region antibody sequence (either full-length or framework only VH, VK, or VL), protein BLAST [19] was performed against all sequences in the respective database. Following BLAST the matching sequences were sorted from high to low based on the percent identity with the input sequence. The percent identity of the Top 20 matched sequences were averaged to obtain the T20 Score (Figure 1B), where the T20 score is a measurement of how human-like the variable region of an antibody looks, with 100 being the highest possible score; most antibodies analyzed with this method scored at least 40. When the T20 score was obtained for each sequence from the curated All Human Databases and then the scores were sorted from high to low, a relatively normal distribution was observed for both full-length and framework only sequences (Figure 2A, Additional file 1: Figure S1A).

An antibody humanness score should possess the ability to distinguish human antibody sequences from other species' antibody sequences, such as mouse, with high specificity. To determine the ability of the T20 score to differentiate human antibody sequences from mouse, we scored thousands of mouse antibody sequences with the T20 score analyzer using the All Human Databases (Figure 2A, Additional file 1: Figure S1A). Most of the scores for human and mouse sequences had clear separation; however some amount of overlap remained between human and mouse VH and VK sequences. One potential reason to explain why many of the mouse sequences are scoring higher T20 scores is due to their similarity with the human sequences that had the lowest T20 scores, i.e. the mouse sequences could be similar to the human sequences that looked the least human-like. If this was correct, we predicted that if the least human-like sequences were removed from the database and the mouse sequences were re-scored, this would create a clearer separation between mouse and human sequences. To test this, we removed the bottom 15% of human sequences from each database to obtain T20 Cutoff Human Databases (Figure 1C). This created a T20 score cutoff, which is the T20 score where sequences above this cutoff are considered human-like. We then re-scored the mouse sequences using the T20 Cutoff Human Databases and found a striking reduction in the T20 scores for the full-length and framework only mouse sequences, with minimal overlap between human and mouse sequences (Figure 2B, Additional file 1: Figure S1B). Importantly, despite removing many human sequences from the databases, re-scoring all human sequences with the refined databases lead to very little change in their T20 scores (Additional file 2: Figure S2). Therefore by developing a humanness analysis tool that focused on the top 85% of human antibody sequences, we were able to clearly separate human and mouse antibody sequences with excellent specificity, and defined human-like sequences as those scoring above the T20 score cutoff. From here on, the T20 score only refers to sequences scored with the T20 Cutoff Human Databases.

### T20 score analysis of a collection of antibody sequences

**Figure 3:**
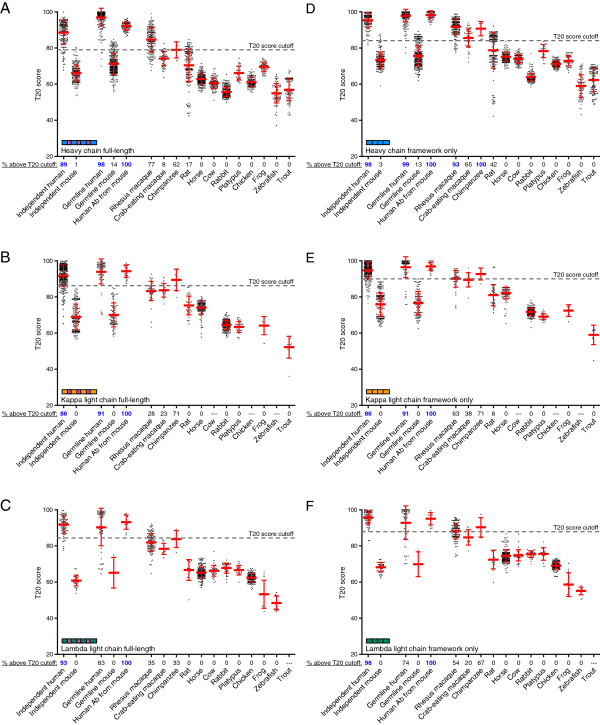
**T20 scores of a collection of antibody sequences.** T20 scores were obtained for each listed group of antibodies for the indicated chain type for full-length antibody sequences **(A**-**C)** or framework only antibody sequences **(D**-**F)**. Individual antibody sequences are shown as small circles, and the average ± SD T20 score is shown for each group. The percent of antibody sequences with T20 scores above their respective T20 score cutoff (indicated by the dashed line) is provided below each graph. The antibody groups with 85% or more of the sequences above the cutoff (i.e. they are mostly human-like) are indicated by the blue text. Groups with dashes for the percent value did not have any sequences represented by the particular chain.

To determine the reproducibility of the T20 score distinguishing most human and mouse sequences based on their variable region sequences, an independent set of human and mouse antibodies were evaluated. For both full-length sequences (Figure 3A-C) and framework only sequences (Figure 3D-F), the T20 score was able to accurately classify the vast majority of human sequences as human-like and most if not all mouse sequences as not human-like. This validated the T20 score as a reproducible method to classify the humanness of an antibody sequence with high specificity.

We next analyzed the variable regions of human and mouse germline sequences, and while there was some variability depending on the chain type being analyzed, in general the T20 score accurately classified the human germline sequences as having high T20 scores with the mouse germline sequences having scores mostly below the human-like T20 score cutoff; similar trends were seen for both full-length and framework only germline sequences (Figure 3). We further found that human antibodies produced in mice that were transgenic for human IgG genes [20-22] produced human antibodies with high T20 scores that were similar to germline sequences. By this metric these human antibodies from mice were indistinguishable from fully human antibodies. Overall, these data further validate the T20 score as a means to consistently and accurately classify antibody sequences as human-like or not.

To determine the humanness of antibody sequences from other species, T20 scores were calculated for full-length and framework only sequences for 12 additional species ranging from fish to non-human primates (Figure 3). Generally, the non-human primate antibody sequences had high T20 scores and thus many of them are difficult to distinguish from human sequences, with the Rhesus macaque and Chimpanzee having the highest scores. Fish sequences showed the lowest T20 scores, while mammals and amphibians were somewhere in between. No antibody sequences from non-primates besides mice and rats looked human-like (Figure 3).

### The humanness of therapeutic antibodies

**Figure 4:**
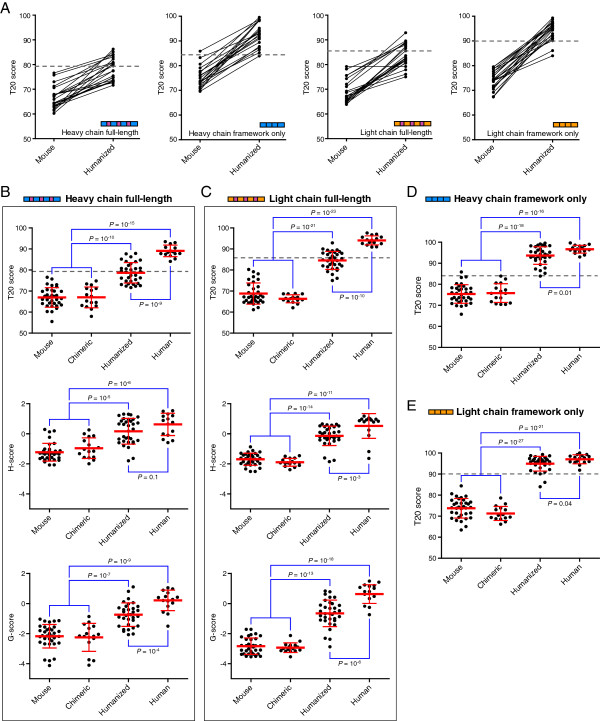
**Humanness of therapeutic antibodies. (A)** T20 scores are shown for the indicated chain type for 22 therapeutic mouse antibodies that underwent humanization. Each circle is one antibody, and the lines connect a mouse antibody with its respective humanized antibody. The T20 score cutoff is indicated by the dashed line. **(B-E)** Shown are the T20 score (top), H-score (middle), and G-score (bottom) of 98 heavy chain full-length **(B)** and kappa light chain full-length **(C)** sequences for mouse, chimeric, humanized, and fully human therapeutic antibodies. Note that the H-score and G-score are scaled based on Z-scores. The T20 score of heavy chain framework only **(D)** and kappa light chain framework only **(E)** sequences are presented. Each antibody is a circle, and the average ± SD T20 score is provided for each group. The T20 score cutoff is indicated by the dashed line. Select groups of antibodies were compared using Student’s t-tests, and the P-values of significance are listed for each comparison.

We curated a set of 98 mouse, chimeric (from mouse), humanized, and fully human antibody sequences that are either approved for clinical use or in various stages of clinical development. We only focused on antibodies with kappa light chain sequences since very few therapeutic antibodies had lambda light chains. We first determined how well the T20 score could detect changes in the variable region sequence of therapeutic antibodies that underwent humanization. Of 22 antibody pairs analyzed, only the light chain of one sequence showed no change in the T20 score while all others showed an increase in their T20 scores upon humanization (Figure 4A). Interestingly, for the full-length sequences after humanization, half of the antibody sequences looked human-like, despite the inclusion of non-human sequences in the CDRs and framework regions. For the framework only sequences, nearly all humanized antibodies had framework sequences that looked human-like. This was generally expected since human germline framework sequences are typically utilized as donor sequences for the humanization process [10], and most germline sequences have very high T20 scores (Figure 3D-E).

We next calculated the average T20 score for each type of therapeutic antibody. As anticipated the humanness of mouse and chimeric antibodies were nearly identical for both full-length and framework only sequences (Figure 4B-E). For full-length sequences, the humanness of humanized heavy and light chain sequences increased strongly, and the T20 score analyzer was sensitive enough to detect a further significant increase in the humanness of fully human antibodies (Figure 4B-C). In contrast for framework only sequences, the humanness of both humanized and human sequences were very similar (Figure 4D-E).

To compare the effectiveness of the T20 score to classify the humanness of antibodies as compared to other antibody humanness scoring tools, we determined the H-score [15] and G-score [17] for the 98 therapeutic antibodies. When the T20 score and H-score or G-score were directly compared for the same antibody sequences, the scores were positively correlated for both heavy and light chain sequences (P<10^-20^ for each comparison; Additional file 3: Figure S3). Despite the positive correlation with the T20 score, the H-score and G-score were unable to separate the humanness of the therapeutic antibody groups as well as the T20 score (Figure 4B-C). In particular the differences between mouse/chimeric and humanized or human antibodies, and the differences between humanized and human sequences, were much less significant with the H-score or G-score as compared to the T20 score. And although the average H-score and G-score for human antibodies were trending higher than humanized antibodies, when individual sequences were analyzed separate from their group, these scoring methods were not able to clearly distinguish most of the humanized antibodies from fully human antibodies to the same extent as the T20 score (Figure 4B-C). These data reveal that the T20 score is more reliable at classifying antibodies as human-like with better specificity than alternative antibody humanness analysis tools.

### Correlation of humanness and immunogenicity of therapeutic antibodies

**Figure 5:**
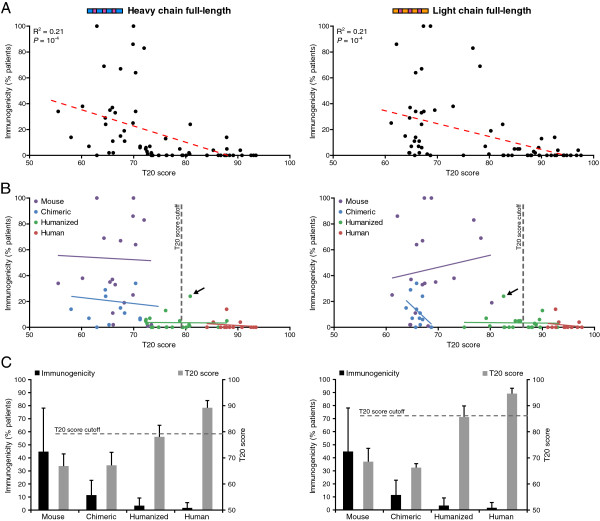
**Associating immunogenicity and humanness score of therapeutic antibodies. (A)** The immunogenicity (percent of patients that had antibodies against the therapeutic antibody itself) and T20 score of 65 therapeutic antibodies (full-length heavy chains on the left, kappa light chains on the right) were graphed together, and Pearson correlations were calculated (red-dashed line; R^2^). P-values are one-sided t-tests. **(B)** The same data from **(A)** is shown with the antibody type indicated by different colors. Trend lines for each group are shown in their respective color. The arrow points to the humanized antibody daclizumab that is referenced in the main text. **(C)** The black bars are the average ± SD immunogenicity of the indicated group of antibodies; the gray bars show the average ± SD T20 score.

Of the 98 therapeutic antibodies curated above, 65 of them had available data on the antibody immunogenicity from human clinical trials ([11,23,24] and references within). When the T20 scores of all therapeutic antibodies were directly compared to their immunogenicity, there was a clear negative correlation for both full-length and framework only sequences: the higher the T20 score, the lower the immunogenicity of the antibody (Figure 5A, Additional file 4: Figure S4A). When these antibodies were separated into the respective antibody class, specific patterns emerged that were similar for full-length and framework only, heavy and light chain sequences. Although mouse and chimeric antibodies had similar T20 scores, on average there was a strong decrease in the immunogenicity of chimeric antibodies compared to mouse (Figure 5B-C, Additional file 4: Figure S4B-C). A further increase in T20 score seen with humanized antibodies resulted in a further drop in the immunogenicity. And although fully human antibodies have an even further increase in T20 score for full-length antibody sequences compared to humanized antibodies, there was little subsequent decrease in their immunogenicity (Figure 5B-C). One exception was the humanized antibody daclizumab that had a strong immunogenic response in 24% of patients (antibody indicated by the arrow in Figure 5B); when this antibody was removed from the analysis, the minor difference in the immunogenicity of humanized and human antibodies disappeared (Additional file 5: Figure S5). These data suggest that because the immunogenicity of humanized and fully human antibodies are nearly identical, with minor exceptions, there may not be an extra benefit of obtaining fully human therapeutic antibodies as long as the humanized antibody has a higher T20 score than its mouse/chimeric parental sequence.

**Figure 6:**
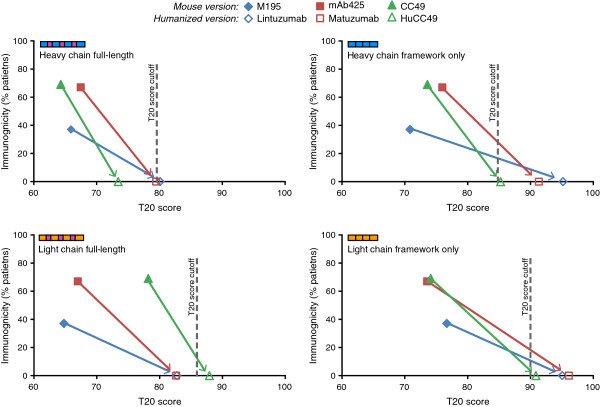
**Immunogenicity of therapeutic antibodies before and after humanization.** The immunogenicity and T20 score of three mouse therapeutic antibodies before and after humanization are shown for the indicated chain type. The arrows show the change in immunogenicity and T20 score of the mouse antibody after humanization.

The benefit of performing antibody humanization was further evidenced by following the humanness and immunogenicity of therapeutic antibodies prior to and after humanization. Within our curated therapeutic antibody database, there were three mouse antibodies that underwent humanization and were included in clinical trials as both mouse and humanized antibodies. All three mouse antibodies initially had high levels of immunogenicity with low T20 scores (Figure 6). The humanness of the antibody heavy and light chain sequences increased following humanization, and this increase correlated with a shift to no detectable immunogenicity in all three cases, underscoring the critical importance of performing antibody humanization prior to conducting clinical trials. The data suggest that the framework only T20 score of the humanized heavy and light chains should be equal to or above the T20 score cutoff (i.e. they are human-like). In contrast, the full-length sequence appears to only need to increase its humanness T20 score, as it does not have to look human-like in order to benefit from antibody humanization and have similar immunogenic properties as a fully human antibody. Therefore quantifying the humanness of therapeutic antibody sequences with the T20 score analyzer is a powerful tool that can be a valuable asset during the antibody drug development process.

## Discussion

A critical issue with therapeutic antibodies in the clinic is reducing the potential immunogenicity of the final therapeutic form [23]. One of the more recent tactics to avoid possible immunogenicity issues is by using fully human antibodies, either produced in transgenic mice [22] or using phage display technology [25], which are often difficult processes compared to traditional monoclonal antibody development [4]. Data presented here revealed that despite a significant difference in the humanness of humanized and fully human antibody sequences, with minor exceptions the immunogenicity of the antibodies was nearly indistinguishable. This was shown in detail for three antibodies that started with high levels of immunogenicity as mouse antibodies, and after their humanization they became non-immunogenic antibodies; the decreased immunogenicity was correlated with an increase in the humanness score of both the heavy and light chains of each antibody. Therefore an additional mechanism to decrease the chance of a humanized antibody being immunogenic in humans is verifying that both the heavy and light chains of the humanized antibody have increased T20 scores over the parental sequence and are thus more human-like. While it is possible that humanized antibodies with lower T20 score will not be immunogenic in the clinic, in general non-human-like antibodies were associated with increased immunogenicity (Figure 5A).

The unique ability of the T20 score analyzer to specifically determine the humanness of the framework regions of an antibody sequence is especially useful when performing antibody humanization, where the CDR sequences are purposely left as parental sequences and only the framework region is transformed into a human-like sequence. For example, the humanness of an antibody framework sequence can be followed throughout the process of performing humanization, where each subsequent change to the framework region can be scored and tracked. Our own antibody humanization efforts revealed that despite the high T20 scores for most donor human germline framework sequences (Figure 3D), re-introducing specific parental framework sequences into a human germline sequence is sometimes sufficient to revert the T20 score of the humanized framework sequence to the same score as the initial parental framework sequence (Additional file 6: Figure S6). Since the T20 score analyzer would be simple to implement during the therapeutic antibody development process, it can be used to prevent antibody sequences with low humanness scores from progressing along the pipeline.

Other methods have been developed to predict antibody immunogenicity, such as experimentally screening molecules for antigen specific activation of T-cells or experimentally searching for T-cell epitopes followed by their removal through targeted substations [23,26]. However these methods require a combination of *in vitro* experimentation in addition to *in silico* prediction tools. We propose that these complicated and time-consuming methods are not necessary since it appears that increasing the humanness of the variable region sequence is sufficient to remove most cases of immunogenicity, which can be directly monitored with the T20 score analyzer.

Researchers have utilized phage display technologies to express synthetic repertoires of antibodies that can be screened for binding to specific antigens [27-29]. During the screening process it would be useful to predict whether these synthetic antibodies would be immunogenic in humans or not, for example by utilizing the T20 score to analyze the sequences. We used the T20 score analyzer to determine the humanness score of a small set of synthetic antibodies and compared these to human antibodies. Surprisingly, the average and range of T20 scores observed for the variable regions of synthetic antibodies was very similar to human antibodies (Additional file 7: Figure S7). Due to the absence of immunogenicity data from synthetic antibodies, we were unable to directly correlate the T20 score of synthetic antibodies with immunogenicity. However since the T20 scores of synthetic and human antibodies are similar, we suggest that the T20 score may be able to predict the immunogenicity of synthetic antibodies to a similar extent as human antibodies.

## Conclusion

Here we have developed the T20 score analyzer to calculate the humanness of variable region sequences of monoclonal antibodies with high specificity and reproducibility. In addition to providing a score for the full-length antibody sequence of heavy, kappa light, and lambda light chains, the tool can exclude the CDR regions to calculate a separate score focusing only on the framework regions. We used this tool to study therapeutic antibodies that have been approved for clinical use or are currently in clinical development. Of note we observed consistent decreases in the immunogenicity of antibodies that underwent humanization that resulted in increased T20 scores, suggesting that the T20 score may be used as a metric to determine whether an antibody has been truly humanized. We further found that the T20 score analyzer was better at assessing the differences in the humanness of therapeutic antibodies compared to previously published humanness scoring methods. This tool will be a valuable asset to accurately measure the humanness of the variable region of new therapeutic antibodies during their preclinical development.

## Methods

### Antibody variable region sequence curation

For the All Human Databases, antibody variable region protein sequences were obtained from NCBI IgBLAST (http://www.ncbi.nlm.nih.gov/igblast/retrieveig.html). Sequences were obtained and processed in high-throughput using scripts written in Python. Variable heavy chain (VH), kappa light chain (VK), and lambda light chain (VL) sequences were downloaded separately. Synthetic Ig molecules were excluded, and the minimum sequence length was set to 90 amino acids. Sequences were assigned Kabat numbering using the Abnum tool [30] and CDR residues were identified following the guidelines put forth by Kabat [18]. Sequences that Abnum was unable to assign the numbering scheme to were excluded from further analysis. Duplicate sequences were removed prior to forming the final databases, and sequences mislabeled as ‘human’ humanized antibodies or human antibodies obtained from transgenic mice were also excluded. In total 29,958 heavy chain sequences, 5,042 kappa light chain sequences, and 3,708 lambda light chain sequences were curated for the All Human Databases.

The mouse protein sequences used to validate the human databases were also obtained from NCBI IgBLAST and processed in the same way as the human sequences. In total 11,781 heavy chain sequences, 3,652 kappa light chain sequences, and 357 lambda light chain sequences were curated.

The human and mouse germline sequences were obtained from NCBI IgBLAST (http://www.ncbi.nlm.nih.gov/igblast/showGermline.cgi). Human antibodies from transgenic mice were selected from the human sequences downloaded from IgBLST described above based on the descriptions found in the NCBI protein database (http://www.ncbi.nlm.nih.gov/protein). The independent set of human and mouse antibody sequences were obtained from Abysis database (http://www.bioinf.org.uk/abysis); these were compared to the sequences in the All Human Databases to remove any overlapping sequences. The antibody sequences for the remaining species and synthetic antibodies were obtained from Abysis and/or NCBI protein database. Sequences for the therapeutic antibodies were obtained from United States patent applications (patft.uspto.gov) and the KEGG drug database (http://www.genome.jp/kegg/drug).

### T20 score analyzer

An input VH, VK, or VL variable region protein sequence is first assigned Kabat numbering and CDR residues are identified. The full-length sequence or the framework only sequence (with CDR residues removed) is compared to every sequence in the respective antibody database using the blastp protein-protein BLAST algorithm [19]. The sequence identity between each pairwise comparison is isolated, and after every sequence in the database has been analyzed, the sequences are sorted from high to low based on the sequence identity to the input sequence. The percent identity of the Top 20 matched sequences is averaged to obtain the T20 score. The T20 score analyzer was coded entirely using Python.

### Formation of T20 cutoff human databases

For each chain type (VH, VK, VL) and sequence length (full-length or framework only) in the All Human Databases, each antibody sequence was scored with its respective database using the T20 score analyzer. The T20 score was obtained for the top 20 matched sequences after the input sequence itself was excluded (the percent identity of sequences 2 through 21 were averaged since sequence 1 was always the input antibody itself). The T20 scores for each group were sorted from high to low. The decrease in score was roughly linear for most of the sequences; however the T20 scores for the bottom ~15% of antibodies started decreasing sharply. Therefore the bottom 15 percent of sequences were removed and the remaining sequences formed the T20 Cutoff Human Databases, where the T20 score cutoff indicates the lowest T20 score of a sequence in the new database. The exact number of antibody sequences in each database for full-length and framework only is provided in Figure 1C; note that the numbers are slightly different for full-length and framework only sequences since the T20 score cutoff was rounded to the nearest whole number closest to the bottom 15th percentile sequence. A web-based tool is provided to calculate the T20 score of antibody sequences using the T20 Cutoff Human Databases: http://abAnalyzer.lakepharma.com.

## Competing interests

All authors are employees of LakePharma, Inc.

## Authors’ contributions

SG curated the antibody databases, co-developed the T20 score analyzer, and calculated the T20 scores for all antibodies in the databases. KH assisted with the design of the T20 score analyzer. HT conceived of the study and provided guidance on the development of the T20 score analyzer. AA co-developed the T20 score analyzer, performed all analyses on the therapeutic antibodies, prepared the figures, and wrote the manuscript. All authors read and approved the manuscript.

## Supplementary Material

Additional file 1: Figure S1 T20 scores distinguish human and mouse antibody framework sequences. **(A)** T20 scores using All Human Databases. Shown are histograms of the T20 scores for large sets of human or mouse antibody sequences of the indicated chain type. The T20 score cutoff for each antibody chain is indicated by the dashed line. **(B)** Comparing scores using T20 All Human and Cutoff Human Databases. Shown are histograms of the T20 scores for the same set of human or mouse antibody sequences scored with the indicated database. Note that the human sequences with scores below the T20 score were removed from these graphs. The percent of mouse antibodies sequences scored with the T20 Cutoff Human Database that are above the T20 cutoff is provided on the right. Click here for file

Additional file 2: Figure S2 Comparing human sequences with All Human and T20 Cutoff Human Databases. Shown are histograms of the T20 scores for all human antibody sequences of the indicated chain type comparing the scores obtained using the indicated scoring database. Human sequences were separated into two groups, either above (red) or below (green) the T20 score cutoff using the All Human Databases. Each of the two groups of sequences was then scored with the T20 Cutoff Human Database, and the change in the T20 scores is shown separately for the sequences above (purple) or below (blue) the T20 score cutoff. Click here for file

Additional file 3: Figure S3 Comparison of T20 score with H-score and G-score. T20 scores, H-scores, and G-scores were obtained for 98 heavy chain full-length (top) and kappa light chain full-length (bottom) therapeutic antibody sequences. Note that the H-score and G-score are scaled based on Z-scores. T20 scores were directly compared to H-scores (left) or G-scores (right), and Pearson correlations were calculated (red-dashed line; R^2^). P-values are one-sided t-tests. Click here for file

Additional file 4: Figure S4 Associating immunogenicity and humanness score of therapeutic antibodies. **(A)** The immunogenicity and T20 score of 65 therapeutic antibodies (framework only heavy chains on the left, kappa light chains on the right) were graphed together, and Pearson correlations were calculated (red-dashed line; R^2^). P-values are one-sided t-tests. **(B)** The same data from (A) is shown, with the antibody type indicated by different colors. Trend lines for each group are shown in their respective color. **(C)** The black bars are the average ± SD immunogenicity of the indicated group of antibodies; the gray bars show the average ± SD T20 score. Click here for file

Additional file 5: Figure S5 No difference in immunogenicity of humanized and human antibodies after removing daclizumab. The black bars are the average ± SD immunogenicity of all human and humanized antibodies; the gray bars show the average ± SD immunogenicity of all human antibodies and humanized antibodies without daclizumab. Click here for file

Additional file 6: Figure S6 Humanization of germline frameworks can revert the humanness of sequences back to the level of the parental antibody. Shown are the T20 scores of a heavy chain antibody that was humanized utilizing donor human germline framework sequences. The parental antibody sequence and three humanized versions with increasing number of re-introduced parental framework amino acids are shown. Note that the humanized version 3 sequence that contains the most parental framework sequence has identical T20 score to the parental sequence, despite the fact that the majority of the humanized sequence is still the human framework sequence. Click here for file

Additional file 7: Figure S7 No difference in T20 score of human antibodies and synthetic antibodies. T20 scores were obtained for each listed group of antibodies for the indicated chain type for full-length antibody sequences. Individual antibody sequences are shown as small circles, and the average ± SD T20 score is shown for each group. Click here for file

## References

[B1] Emmons C Hunsicker LG Muromonab-CD3 (Orthoclone OKT3): the first monoclonal antibody approved for therapeutic use Iowa Med 1987 77 2 78 82 3557906

[B2] Reichert JM Marketed therapeutic antibodies compendium MAbs 2012 4 3 413 415 22531442 10.4161/mabs.19931 PMC3355480

[B3] Piggee C Therapeutic antibodies coming through the pipeline Anal Chem 2008 80 7 2305 2310 10.1021/ac086033v 18456912

[B4] Nelson AL Dhimolea E Reichert JM Development trends for human monoclonal antibody therapeutics Nat Rev Drug Discov 2010 9 10 767 774 10.1038/nrd3229 20811384

[B5] Chan AC Carter PJ Therapeutic antibodies for autoimmunity and inflammation Nat Rev Immunol 2010 10 5 301 316 10.1038/nri2761 20414204

[B6] Weiner LM Surana R Wang S Monoclonal antibodies: versatile platforms for cancer immunotherapy Nat Rev Immunol 2010 10 5 317 327 10.1038/nri2744 20414205 PMC3508064

[B7] Reichert JM Which are the antibodies to watch in 2012? MAbs 2012 4 1 1 3 10.4161/mabs.4.1.18719 22327425 PMC3338935

[B8] Morrison SL Johnson MJ Herzenberg LA Oi VT Chimeric human antibody molecules: mouse antigen-binding domains with human constant region domains Proc Natl Acad Sci USA 1984 81 21 6851 6855 10.1073/pnas.81.21.6851 6436822 PMC392030

[B9] Jones PT Dear PH Foote J Neuberger MS Winter G Replacing the complementarity-determining regions in a human antibody with those from a mouse Nature 1986 321 6069 522 525 10.1038/321522a0 3713831

[B10] Almagro JC Fransson J Humanization of antibodies Front Biosci 2008 13 1619 1633 17981654 10.2741/2786

[B11] Hwang WY Foote J Immunogenicity of engineered antibodies Methods 2005 36 1 3 10 10.1016/j.ymeth.2005.01.001 15848070

[B12] Hansel TT Kropshofer H Singer T Mitchell JA George AJ The safety and side effects of monoclonal antibodies Nat Rev Drug Discov 2010 9 4 325 338 10.1038/nrd3003 20305665

[B13] Chirino AJ Ary ML Marshall SA Minimizing the immunogenicity of protein therapeutics Drug Discov Today 2004 9 2 82 90 10.1016/S1359-6446(03)02953-2 15012932

[B14] Wu TT Kabat EA An analysis of the sequences of the variable regions of Bence Jones proteins and myeloma light chains and their implications for antibody complementarity J Exp Med 1970 132 2 211 250 10.1084/jem.132.2.211 5508247 PMC2138737

[B15] Abhinandan KR Martin AC Analyzing the "degree of humanness" of antibody sequences J Mol Biol 2007 369 3 852 862 10.1016/j.jmb.2007.02.100 17442342

[B16] Pelat T Bedouelle H Rees AR Crennell SJ Lefranc MP Thullier P Germline humanization of a non-human primate antibody that neutralizes the anthrax toxin, by in vitro and in silico engineering J Mol Biol 2008 384 5 1400 1407 10.1016/j.jmb.2008.10.033 18976662

[B17] Thullier P Huish O Pelat T Martin AC The humanness of macaque antibody sequences J Mol Biol 2010 396 5 1439 1450 10.1016/j.jmb.2009.12.041 20043919

[B18] Kabat EA Wu TT Reid-Miller M Perry HM Gottesman KS Sequences of Proteins of Immunological Interest 1991 Washington, DC: National Institutes of Health

[B19] Altschul SF Madden TL Schaffer AA Zhang J Zhang Z Miller W Lipman DJ Gapped BLAST and PSI-BLAST: a new generation of protein database search programs Nucleic Acids Res 1997 25 17 3389 3402 10.1093/nar/25.17.3389 9254694 PMC146917

[B20] Suarez E Magadan S Sanjuan I Valladares M Molina A Gambon F Diaz-Espada F Gonzalez-Fernandez A Rearrangement of only one human IGHV gene is sufficient to generate a wide repertoire of antigen specific antibody responses in transgenic mice Mol Immunol 2006 43 11 1827 1835 10.1016/j.molimm.2005.10.015 16343622

[B21] Protopapadakis E Kokla A Tzartos SJ Mamalaki A Isolation and characterization of human anti-acetylcholine receptor monoclonal antibodies from transgenic mice expressing human immunoglobulin loci Eur J Immunol 2005 35 6 1960 1968 10.1002/eji.200526173 15915538

[B22] Jakobovits A Production and selection of antigen-specific fully human monoclonal antibodies from mice engineered with human Ig loci Adv Drug Deliv Rev 1998 31 1–2 33 42 10837616 10.1016/s0169-409x(97)00092-6

[B23] Baker MP Reynolds HM Lumicisi B Bryson CJ Immunogenicity of protein therapeutics: The key causes, consequences and challenges Self/nonself 2010 1 4 314 322 10.4161/self.1.4.13904 21487506 PMC3062386

[B24] Harding FA Stickler MM Razo J DuBridge RB The immunogenicity of humanized and fully human antibodies: residual immunogenicity resides in the CDR regions MAbs 2010 2 3 256 265 10.4161/mabs.2.3.11641 20400861 PMC2881252

[B25] McCafferty J Griffiths AD Winter G Chiswell DJ Phage antibodies: filamentous phage displaying antibody variable domains Nature 1990 348 6301 552 554 10.1038/348552a0 2247164

[B26] Jones TD Crompton LJ Carr FJ Baker MP Deimmunization of monoclonal antibodies Methods Mol Biol 2009 525 405 423 xiv 10.1007/978-1-59745-554-1_21 19252848

[B27] Sidhu SS Li B Chen Y Fellouse FA Eigenbrot C Fuh G Phage-displayed antibody libraries of synthetic heavy chain complementarity determining regions J Mol Biol 2004 338 2 299 310 10.1016/j.jmb.2004.02.050 15066433

[B28] Knappik A Ge L Honegger A Pack P Fischer M Wellnhofer G Hoess A Wolle J Pluckthun A Virnekas B Fully synthetic human combinatorial antibody libraries (HuCAL) based on modular consensus frameworks and CDRs randomized with trinucleotides J Mol Biol 2000 296 1 57 86 10.1006/jmbi.1999.3444 10656818

[B29] Barbas CF 3rd Bain JD Hoekstra DM Lerner RA Semisynthetic combinatorial antibody libraries: a chemical solution to the diversity problem Proc Natl Acad Sci USA 1992 89 10 4457 4461 10.1073/pnas.89.10.4457 1584777 PMC49101

[B30] Abhinandan KR Martin AC Analysis and improvements to Kabat and structurally correct numbering of antibody variable domains Mol Immunol 2008 45 14 3832 3839 10.1016/j.molimm.2008.05.022 18614234

