# Supplementary material for: Monoclonal antibody humanness score and its applications
Source: BMC Biotechnol. 2013 Jul 5;13:55. doi: 10.1186/1472-6750-13-55 (PMC3729710; doi:10.1186/1472-6750-13-55)
Supplement: Additional file 1: Figure S1 — T20 scores distinguish human and mouse antibody framework sequences. (A) T20 scores using All Human Databases. Shown are histograms of the T20 scores for large sets of human or mouse antibody sequences of the indicated chain type. The T20 score cutoff for each antibody chain is indicated by the dashed line. (B) Comparing scores using T20 All Human and Cutoff Human Databases. Shown are histograms of the T20 scores for the same set of human or mouse antibody sequences scored with the indicated database. Note that the human sequences with scores below the T20 score were removed from these graphs. The percent of mouse antibodies sequences scored with the T20 Cutoff Human Database that are above the T20 cutoff is provided on the right. [file 1472-6750-13-55-S1.pdf]

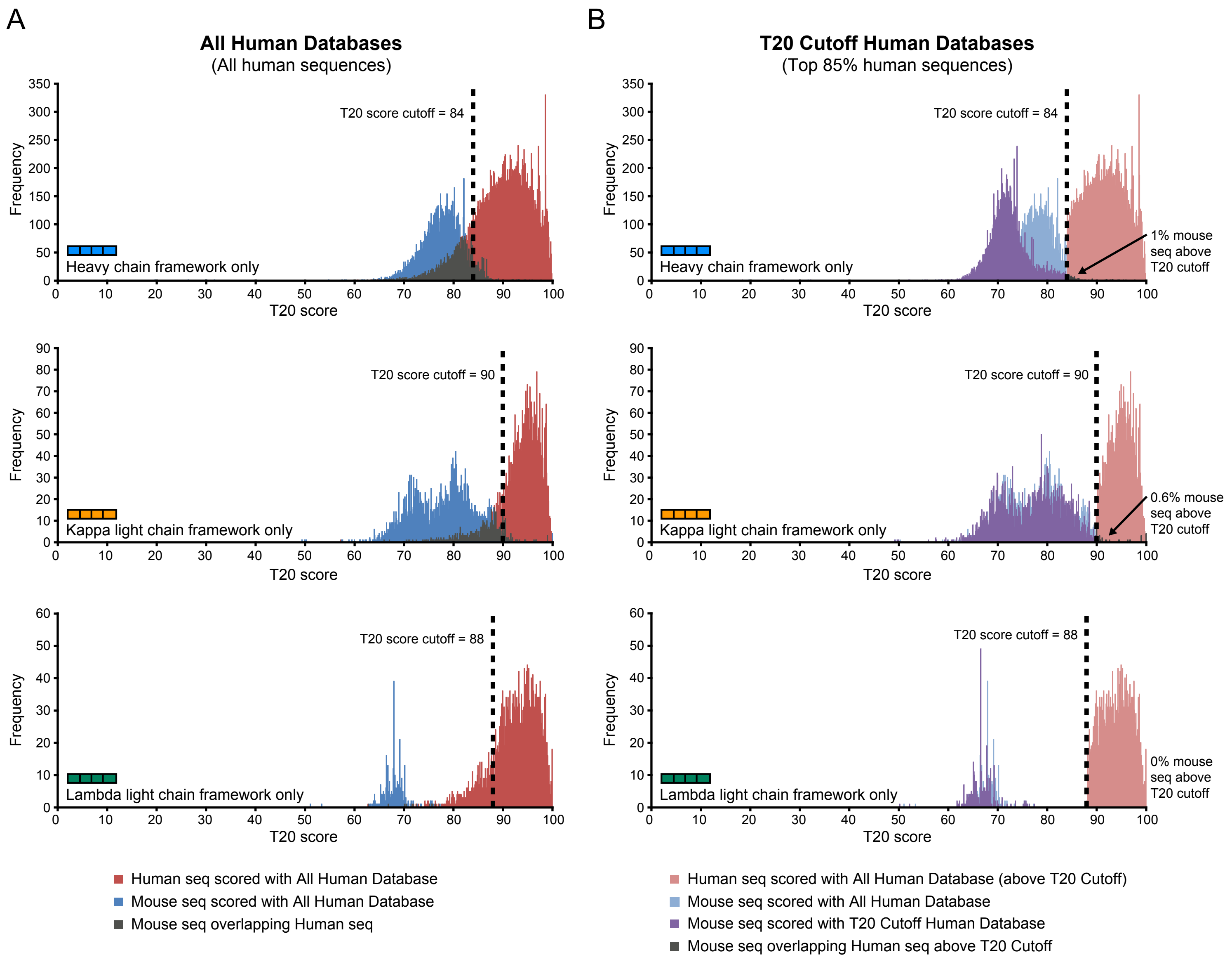


**Figure S1 T20 scores distinguish human and mouse antibody framework sequences.** (**A**) T20 scores using All Human Databases. Shown are histograms of the T20 scores for large sets of human or mouse antibody sequences of the indicated chain type. The T20 score cutoff for each antibody chain is indicated by the dashed line. (**B**) Comparing scores using T20 All Human and Cutoff Human Databases. Shown are histograms of the T20 scores for the same set of human or mouse antibody sequences scored with the indicated database. Note that the human sequences with scores below the T20 score were removed from these graphs. The percent of mouse antibodies sequences scored with the T20 Cutoff Human Database that are above the T20 cutoff is provided on the right.
