# Supplementary material for: Monoclonal antibody humanness score and its applications
Source: BMC Biotechnol. 2013 Jul 5;13:55. doi: 10.1186/1472-6750-13-55 (PMC3729710; doi:10.1186/1472-6750-13-55)
Supplement: Additional file 2: Figure S2 — Comparing human sequences with All Human and T20 Cutoff Human Databases. Shown are histograms of the T20 scores for all human antibody sequences of the indicated chain type comparing the scores obtained using the indicated scoring database. Human sequences were separated into two groups, either above (red) or below (green) the T20 score cutoff using the All Human Databases. Each of the two groups of sequences was then scored with the T20 Cutoff Human Database, and the change in the T20 scores is shown separately for the sequences above (purple) or below (blue) the T20 score cutoff. [file 1472-6750-13-55-S2.pdf]

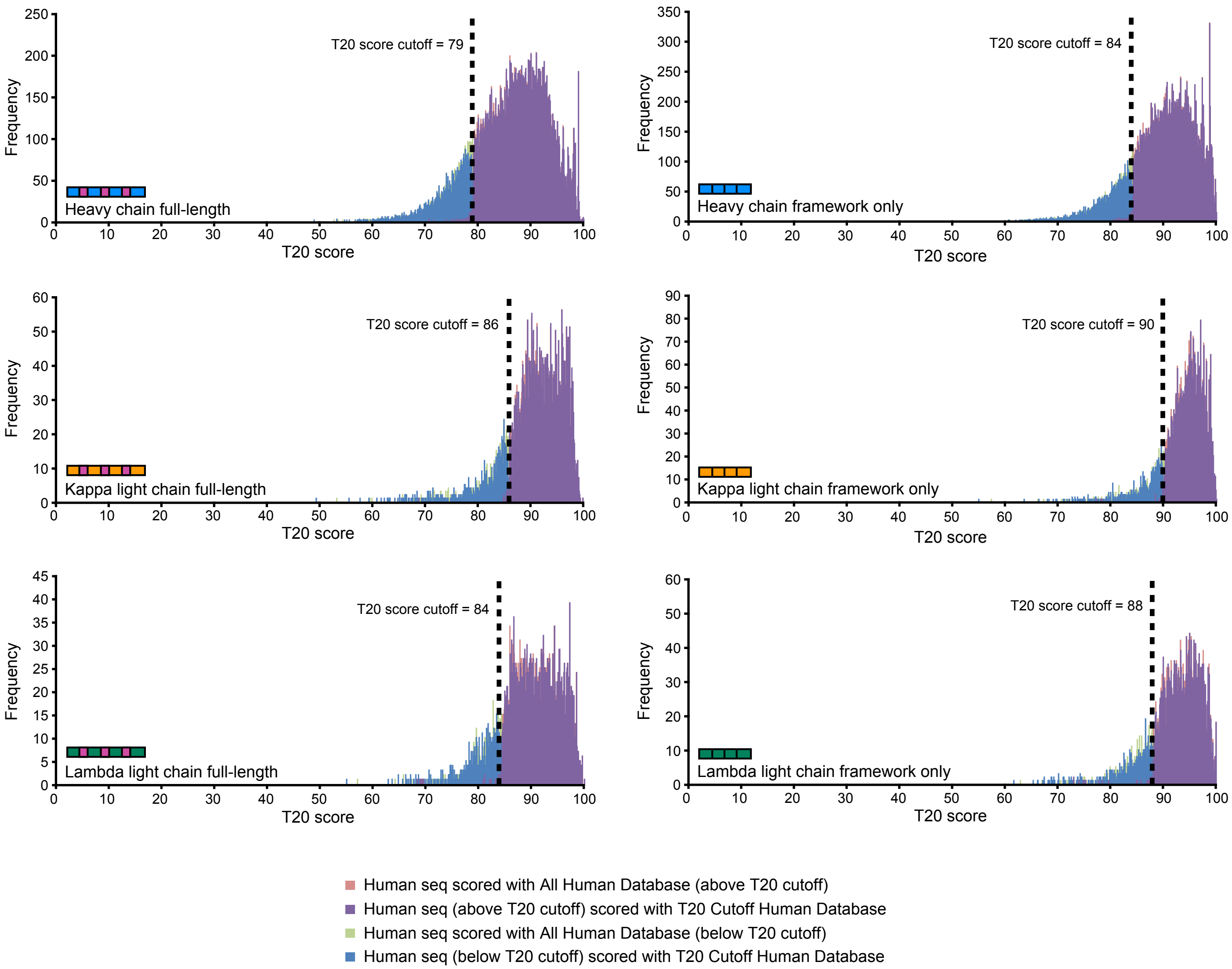

Figure S2 Comparing human sequences with All Human and T20 Cutoff Human Databases. Shown are histograms of the T20 scores for all human antibody sequences of the indicated chain type comparing the scores obtained using the indicated scoring database. Human sequences were separated into two groups, either above (red) or below (green) the T20 score cutoff using the All Human Databases. Each of the two groups of sequences was then scored with the T20 Cutoff Human Database, and the change in the T20 scores is shown separately for the sequences above (purple) or below (blue) the T20 score cutoff.
