# Supplementary material for: Monoclonal antibody humanness score and its applications
Source: BMC Biotechnol. 2013 Jul 5;13:55. doi: 10.1186/1472-6750-13-55 (PMC3729710; doi:10.1186/1472-6750-13-55)
Supplement: Additional file 3: Figure S3 — Comparison of T20 score with H-score and G-score. T20 scores, H-scores, and G-scores were obtained for 98 heavy chain full-length (top) and kappa light chain full-length (bottom) therapeutic antibody sequences. Note that the H-score and G-score are scaled based on Z-scores. T20 scores were directly compared to H-scores (left) or G-scores (right), and Pearson correlations were calculated (red-dashed line; R2). P-values are one-sided t-tests. [file 1472-6750-13-55-S3.pdf]

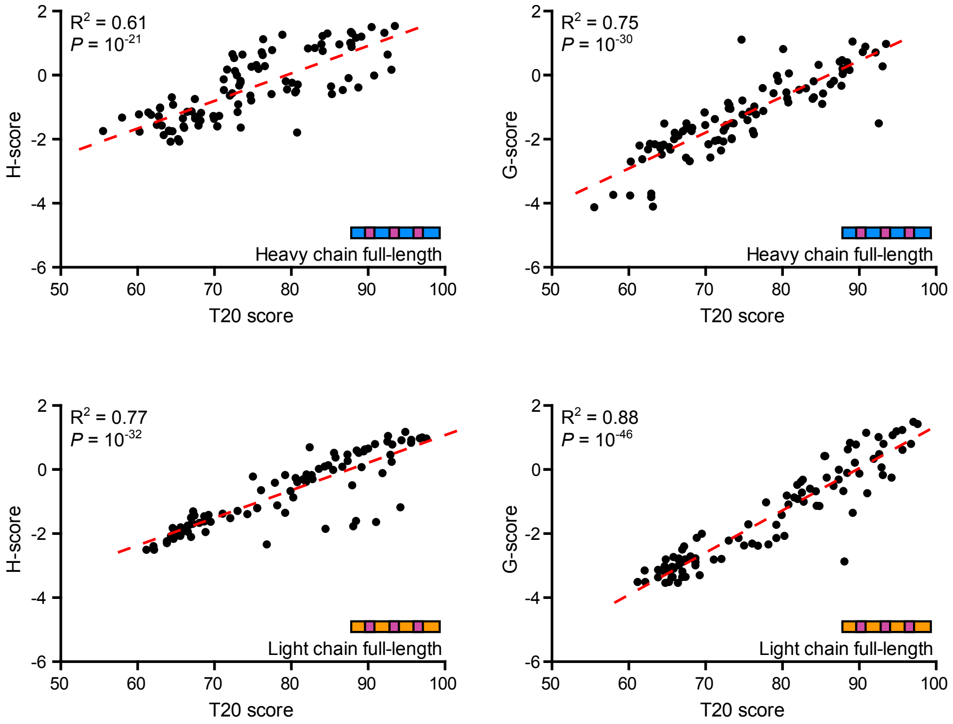

Figure S3 Comparison of T20 score with H-score and G-score. T20 scores, H-scores, and G-scores were obtained for 98 heavy chain full-length (top) and kappa light chain full-length (bottom) therapeutic antibody sequences. Note that the H-score and G-score are scaled based on Z-scores. T20 scores were directly compared to H-scores (left) or G-score (right), and Pearson correlations were calculated (red-dashed line; R^2). *P*-values are one-sided *t*-tests.
