# Supplementary material for: Monoclonal antibody humanness score and its applications
Source: BMC Biotechnol. 2013 Jul 5;13:55. doi: 10.1186/1472-6750-13-55 (PMC3729710; doi:10.1186/1472-6750-13-55)
Supplement: Additional file 4: Figure S4 — Associating immunogenicity and humanness score of therapeutic antibodies. (A) The immunogenicity and T20 score of 65 therapeutic antibodies (framework only heavy chains on the left, kappa light chains on the right) were graphed together, and Pearson correlations were calculated (red-dashed line; R2). P-values are one-sided t-tests. (B) The same data from (A) is shown, with the antibody type indicated by different colors. Trend lines for each group are shown in their respective color. (C) The black bars are the average ± SD immunogenicity of the indicated group of antibodies; the gray bars show the average ± SD T20 score. [file 1472-6750-13-55-S4.pdf]

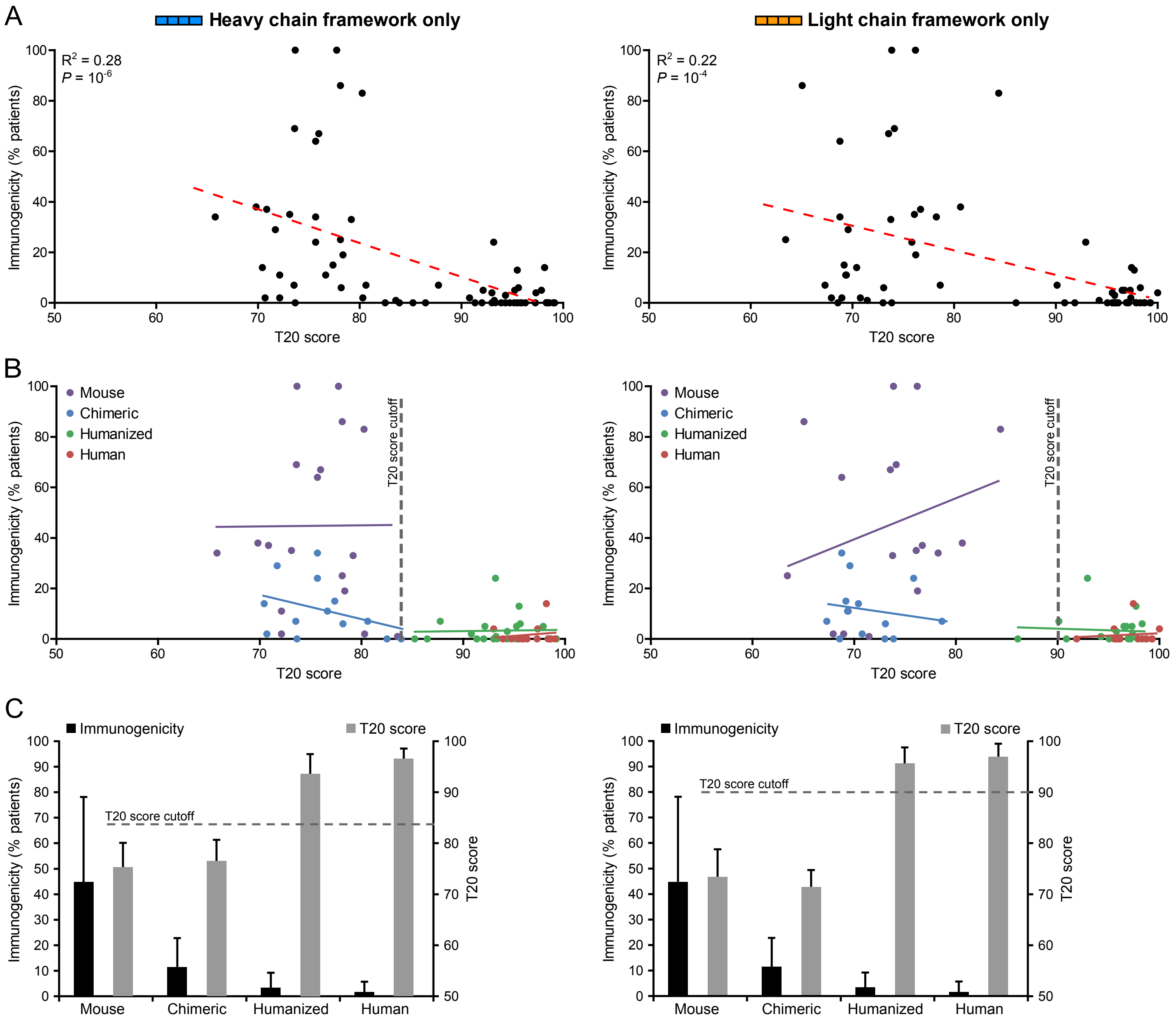

Figure S4 Associating immunogenicity and humanness score of therapeutic antibodies. (**A**) The immunogenicity and T20 score of 65 therapeutic antibodies (framework only heavy chains on the left, kappa light chains on the right) were graphed together, and Pearson correlations were calculated (red-dashed line; R^2). *P*-values are one-sided *t*-tests. (**B**) The same data from (A) is shown, with the antibody type indicated by different colors. Trend lines for each group are shown in their respective color. (**C**) The black bars are the average ± SD immunogenicity of the indicated group of antibodies; the gray bars show the average ± SD T20 score.
