# Supplementary material for: Monoclonal antibody humanness score and its applications
Source: BMC Biotechnol. 2013 Jul 5;13:55. doi: 10.1186/1472-6750-13-55 (PMC3729710; doi:10.1186/1472-6750-13-55)
Supplement: Additional file 5: Figure S5 — No difference in immunogenicity of humanized and human antibodies after removing daclizumab. The black bars are the average ± SD immunogenicity of all human and humanized antibodies; the gray bars show the average ± SD immunogenicity of all human antibodies and humanized antibodies without daclizumab. [file 1472-6750-13-55-S5.pdf]

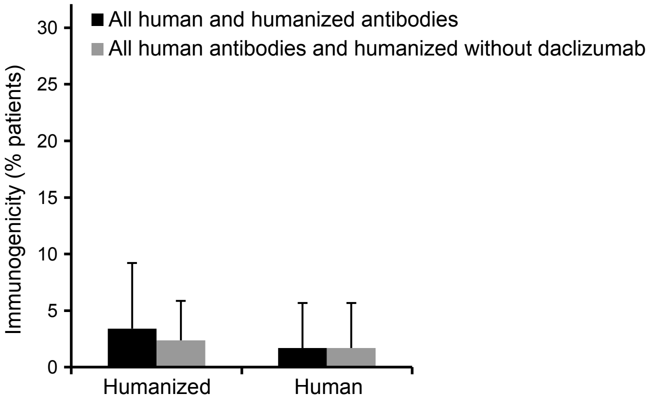

Figure S5 No difference in immunogenicity of humanized and human antibodies after removing daclizumab. The black bars are the average ± SD immunogenicity of all human and humanized and antibodies; the gray bars show the average ± SD immunogenicity of and all human antibodies and humanized antibodies without daclizumab.
