# Supplementary material for: Monoclonal antibody humanness score and its applications
Source: BMC Biotechnol. 2013 Jul 5;13:55. doi: 10.1186/1472-6750-13-55 (PMC3729710; doi:10.1186/1472-6750-13-55)
Supplement: Additional file 6: Figure S6 — Humanization of germline frameworks can revert the humanness of sequences back to the level of the parental antibody. Shown are the T20 scores of a heavy chain antibody that was humanized utilizing donor human germline framework sequences. The parental antibody sequence and three humanized versions with increasing number of re-introduced parental framework amino acids are shown. Note that the humanized version 3 sequence that contains the most parental framework sequence has identical T20 score to the parental sequence, despite the fact that the majority of the humanized sequence is still the human framework sequence. [file 1472-6750-13-55-S6.pdf]

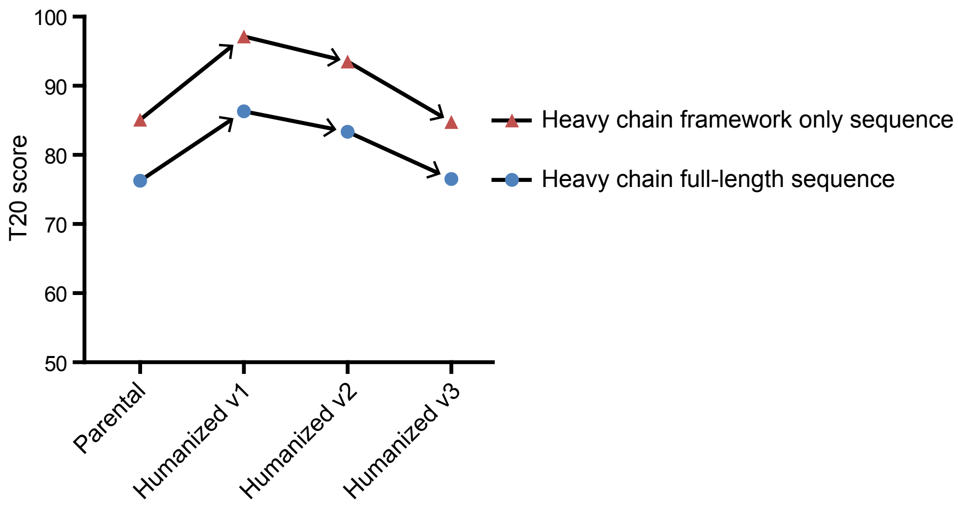

Figure S6 Humanization of germline frameworks can revert the humanness of sequences back to the level of the parental antibody. Shown are the T20 scores of a heavy chain antibody that was humanized utilizing donor human germline framework sequences. The parental antibody sequence and three humanized versions with increasing number of re-introduced parental framework amino acids are shown. Note that the humanized version 3 sequence that contains the most parental framework sequence has identical T20 score to the parental sequence, despite the fact that the majority of the humanized sequence is still the human framework sequence.
