# Supplementary material for: Monoclonal antibody humanness score and its applications
Source: BMC Biotechnol. 2013 Jul 5;13:55. doi: 10.1186/1472-6750-13-55 (PMC3729710; doi:10.1186/1472-6750-13-55)
Supplement: Additional file 7: Figure S7 — No difference in T20 score of human antibodies and synthetic antibodies. T20 scores were obtained for each listed group of antibodies for the indicated chain type for full-length antibody sequences. Individual antibody sequences are shown as small circles, and the average ± SD T20 score is shown for each group. [file 1472-6750-13-55-S7.pdf]

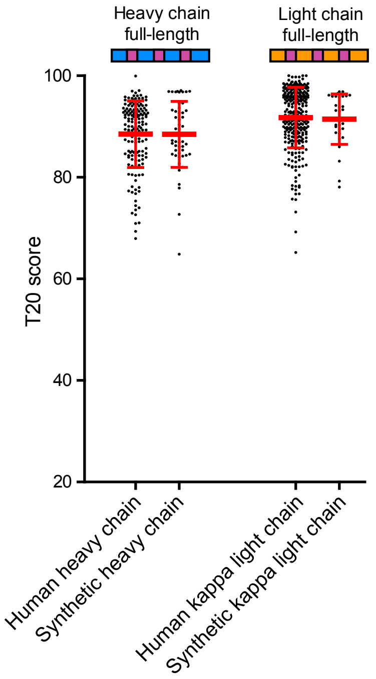

Figure S7 No difference in T20 score of human antibodies and synthetic antibodies. T20 scores were obtained for each listed group of antibodies for the indicated chain type for full-length antibody sequences. Individual antibody sequences are shown as small circles, and the average ± SD T20 score is shown for each group.
